# Some Unusual Affections of the Vermiform Appendix

**Published:** 1909-10-30

**Authors:** 


### SOME UNUSUAL AFFECTIONS OF THE VERMIFORM APPENDIX.

Pyogenic inflammations—the result of the activity of the numerous cocci and bacilli which inhabit, or may inhabit, the cæcum—constitute such an overwhelming proportion of the cases of appendicitis met with in practice, that other affections of which it is occasionally the seat do not receive the attention their importance deserves.

*Primary tuberculosis* of the appendix occurs not infrequently. In its early stages there is nothing which distinguishes it—at any rate in our present state of knowledge—from an ordinary attack of appendicitis of subacute character, but it may be suspected when a thickened, not very tender mass, persists without elevation of pulse or temperature. On removal the appendix often has no special features beyond those of chronic inflammatory thickening—the true pathology of the condition being revealed by histological and bacteriological investigation.

In its later stages, when the whole of the cæcum and surrounding tissues have been converted into a large infiltrated mass of tubercle, the condition becomes more obvious—although the differential diagnosis between inflammation and new growth is not always easy even with the belly open.

*Actinomycosis* is another chronic inflammatory affection which may primarily affect the appendix; but here, owing to the more rapid march of events, the number of cases in which actinomycotic process has indubitably originated in the appendix is very small. This condition is of special interest, owing to the mode of progress of the disease, for actinomycosis knows no anatomical boundaries: it infiltrates mucous membrane, muscle, or even bone without hesitation, with the result that before very long it inevitably reaches the surface by one or more tracks and discharges its granule-laden pus through multiple fistulous openings which may be found as high up as the loin or low down upon the buttock; and even direct invasion of the bladder is not unknown. In a few cases the inflammatory process spreads in exactly the same way as some acute retro-colic abscesses of appendical origin, and extending through the diaphragm gives rise to direct infection of the pleural sac or the lung.

An added danger of actinomycosis, whether originating here or elsewhere, is the tendency to metastasis by way of the blood-stream; leading to the formation of secondary abscesses in the lungs, liver, or brain.

Surgery can do but little for these advanced cases. Sinuses may be scraped and very large doses of potass. iodid. may be administered (ʒj-ʒij daily), but the prognosis is very grave, for iodides do not appear to have the same—almost specific—action on the abdominal cases as in actinomycosis of the cervical glands. In early cases, when the ray fungus is confined to the appendix, the removal of the organ will bring about a cure.

*Malignant growth* arising in the appendix should of course be columnar cell carcinoma—and here one is not on such certain ground. Cases of carcinoma of the appendix have been reported—but it is noteworthy that in the majority of instances the tumour has been found more or less by accident—either at operation for other abdominal conditions or in the post-mortem room; in no less than 62 per cent. of the cases the patients were under thirty years of age; and the neoplasms in many instances did not conform to type.

However, when carcinoma occurs in other situations in young people, it not infrequently departs from the usual type; and although some of the cases described as carcinoma are possibly instances of hyperplasia of the Lieberkühn's follicles of simple nature, in other cases the histological picture more nearly approaches that of adeno-carcinoma of ordinary character. Systematic histological examination of all appendices removed may prove that carcinoma is a more common condition than the list of only 69 recorded cases would at present indicate.

It is possible that in some of the cases in which the cæcum and the viscera in its immediate neighbourhood are apparently the seat of an extensive carcinomatous mass, careful dissection may indicate the appendix as the site of origin of the growth. In dealing with this particular class of case, however, it is very important to remember that many of these so-called "carcinomatous" masses have ultimately turned out to be either hyperplastic tuberculosis or even chronic actinomycosis—a fact which helps to explain the complete recovery sometimes occurring after the abdomen has been explored and the patient sent back to bed as "inoperable and hopeless."

*Typhoid ulcers* may occur in the appendix, just as they may occur in the cæcum, but owing to the anatomical structure and the narrow lumen of the appendix, their presence may give rise to all the symptoms of acute appendicitis.

The general view is that there is no more justification for the removal of an appendix in such a condition than for the excision of the inflamed Peyers' patches in the small intestine. In the case of perforation the treatment would, of course, be identical with that adopted for the rupture of a typhoid lesion elsewhere in the bowel—prompt laparotomy.

Similarly in *amœbic dysentery*, the appendix may become the seat of the typical lesions—an awkward complication should the ingenious operation of appendicostomy ever be extended to the treatment of acute dysentery.

